# Mediated Electron Transfer at Vertically Aligned Single-Walled Carbon Nanotube Electrodes During Detection of DNA Hybridization

**DOI:** 10.1186/s11671-015-0978-0

**Published:** 2015-06-24

**Authors:** Rachel Wallen, Nirmal Gokarn, Priscila Bercea, Elissa Grzincic, Krisanu Bandyopadhyay

**Affiliations:** Department of Natural Sciences, University of Michigan-Dearborn, 4901 Evergreen Road, Dearborn, MI 48128 USA

**Keywords:** Atomic force microscopy (AFM), Cyclic voltammetry, Electron transfer, Impedance spectroscopy, Single-walled carbon nanotube

## Abstract

Vertically aligned single-walled carbon nanotube (VASWCNT) assemblies are generated on cysteamine and 2-mercaptoethanol (2-ME)-functionalized gold surfaces through amide bond formation between carboxylic groups generated at the end of acid-shortened single-walled carbon nanotubes (SWCNTs) and amine groups present on the gold surfaces. Atomic force microscopy (AFM) imaging confirms the vertical alignment mode of SWCNT attachment through significant changes in surface roughness compared to bare gold surfaces and the lack of any horizontally aligned SWCNTs present. These SWCNT assemblies are further modified with an amine-terminated single-stranded probe-DNA. Subsequent hybridization of the surface-bound probe-DNA in the presence of complementary strands in solution is followed using impedance measurements in the presence of Fe(CN)_6_^3−/4−^ as the redox probe in solution, which show changes in the interfacial electrochemical properties, specifically the charge-transfer resistance, due to hybridization. In addition, hybridization of the probe-DNA is also compared when it is attached directly to the gold surfaces without any intermediary SWCNTs. Contrary to our expectations, impedance measurements show a decrease in charge-transfer resistance with time due to hybridization with 300 nM complementary DNA in solution with the probe-DNA attached to SWCNTs. In contrast, an increase in charge-transfer resistance is observed with time during hybridization when the probe-DNA is attached directly to the gold surfaces. The decrease in charge-transfer resistance during hybridization in the presence of VASWCNTs indicates an enhancement in the electron transfer process of the redox probe at the VASWCNT-modified electrode. The results suggest that VASWCNTs are acting as mediators of electron transfer, which facilitate the charge transfer of the redox probe at the electrode–solution interface.

## Background

Significant progress has been made over the last few years to generate vertically aligned single-walled carbon nanotube (VASWCNT) arrays on various surfaces due to their potential applications in nanoscale circuits [1]; ultrathin, flexible, and transparent conductors [2]; super-capacitors [3]; and, in particular, novel biosensors [4, 5]. One of the main approaches that has evolved over the years to vertical assembly of single-walled carbon nanotubes (SWCNTs) relies on chemical self-assembly of acid-shortened SWCNTs [6, 7]. VASWCNT arrays generated by self-assembly techniques are particularly attractive because of the low-temperature wet-chemical approach. Due to their range of electrical conductivities, excellent chemical stability, high surface area, and tubular structure, VASWCNTs have attracted much attention in order to achieve high sensitivity and detection limits in electrochemical biosensors [8]. This has also been exemplified in a number of recent studies on the advantages of facile electron transfer properties of VASWCNTs compared to randomly distributed, disordered SWCNT mats on electrode surfaces [8–12]. In addition to the limited number of reports on post-synthesis assembly, the electrochemistry of these systems is also not yet fully understood. Fundamental electron and charge-transfer processes associated with these electrodes in the presence of a redox probe in solution are particularly significant in the fabrication of any novel electrochemical biosensor. Although recent literature has shown increased applications of SWCNT arrays as amperometric and voltammetric biosensors [8], only very few reports are available on the electron transfer mechanism of these SWCNT assemblies. Diao et al. [13] have demonstrated quasireversible cyclic voltammetric (CV) behavior of a redox couple in solution for SWCNT arrays generated by coupling with an insulating monolayer on a gold electrode, suggesting electron exchange between the underlying gold electrode and the redox couple in solution, with the possibility of through-bond tunneling between the gold electrode and SWCNTs. In another report, Huang et al. [14] suggested a Randles equivalent circuit [15] to explain slow electron transfer in the electrodes, and they proposed that a large number of SWCNTs act as individual nanoelectrodes. Electrochemical impedance spectroscopy (EIS), an alternating current (AC)-based technique, has been used to study a variety of electrochemical phenomena over wide frequency ranges to investigate the electron and charge-transfer processes occurring at the electrode [14]. Recently, EIS has also emerged as an effective tool to follow DNA hybridization and is particularly promising for its simple, rapid, and low-cost detection of biomolecules [15]. The use of the impedance technique aids fundamental understanding of the electrochemical process at VASWCNT electrodes.

In this report, VASWCNTs are generated on gold surfaces using the self-assembly approach to study electron transfer properties in order to assess these arrays as potential DNA biosensor platforms. The present study is motivated by recent literature reports that VASWCNTs act as molecular wires to promote electrical communication between the underlying electrode and a redox enzyme (including those where the redox center is embedded deep within the glycoprotein shell) [11, 16]. Such direct electron transfer between the enzyme and an electrode surface eliminates the need for redox mediators and thus is extremely attractive for developing *label-free* sensing devices. VASWCNT arrays are created on an amine-functionalized gold surface by introduction of carboxyl groups at the ends of acid-shortened SWCNTs followed by coupling of carboxyl groups with the surface amine moiety through carbodiimide chemistry [11]. In the next step, an amine-terminated DNA is coupled to the free end of the aligned SWCNTs. The subsequent hybridization of the surface-bound probe-DNA with the complementary DNA strands in solution is followed using EIS. Hybridization with the probe-DNA attached to the SWCNTs is expected to change the surface charge of the electrode, which can be monitored by measuring the change in surface resistance using frequency-dependent impedance measurement [17, 18]. In our case, [Fe(CN)_6_]^3−/4−^ is used as the redox probe to report the change in interfacial properties associated with DNA hybridization. For comparison, DNA hybridization in the presence of the redox probe is also followed when the probe-DNA is attached directly to the gold surfaces. In contrast to our expectation of increasing charge-transfer resistance due to repulsion of the negatively charged redox probe by anionic DNA during hybridization, the charge-transfer resistance decreases with time during hybridization when the probe-DNA is attached to VASWCNTs. However, the expected increase in charge-transfer resistance is observed with time during hybridization of the probe-DNA attached directly to the gold surfaces. These results are rationalized by considering the mediated electron transfer through VASWCNTs. The significance of the present study is the generation of VASWCNTs in conjunction with EIS to achieve fundamental understanding of the electron transfer process and eventually use the SWCNT array platform to detect DNA hybridization by applying EIS as the detection technique, which may lead to a label-free biosensor. This methodology of DNA detection can further be extended to detect other biomolecules such as proteins and antibodies.

## Methods

### Materials

In the present work, two probe strands, a target strand [19] and a non-complementary strand of custom-synthesized oligonucleotide, were used. The probe strand used for attaching DNA to SWCNTs had the sequence 5′-NH_2_-C_6_-AGGCTCCTCGCGCACT-3′, containing an amino group and a six-carbon chain at the 5′ end of the DNA. The target strand sequence was 5′-AGT GCG CGA GGA GCC T-3′, and the non-complementary strand had the sequence 5′-GTG CTC CTC TCG CAC G-3′ [19]. The probe strand used when attaching DNA directly to the gold electrode had the same sequence (5′-SH-C_6_-AGGCTCCTGGCG CACT-3′), apart from having a thiol instead of an amino group at the 5′ end for SWCNTs. All DNAs were obtained from Integrated DNA Technologies Inc. (Coralville, IA). Concentrated sulfuric acid, 50–70 % nitric acid, and concentrated hydrochloric acid were obtained from Fisher Scientific (Pittsburgh, PA). Ethanol (200 proof, absolute) (99.5 %), cysteamine (95 %), and D (99.0+ %) were all obtained from Sigma-Aldrich (St. Louis, MO) and used as received. 2-Mercaptoethanol (2-ME) was obtained from J.T. Baker Chemical (Center Valley, PA), and SWCNTs were obtained from Carbon Nanotechnologies, Inc. (now Unidym) (Sunnyvale, CA), which were generated using a high-pressure carbon monoxide (HiPco) method. The sample was the “Pure” category of the SWCNT product out of three different types of SWCNT products available based on the level of purity. SWCNTs were received as dry black powder form with individual SWCNT diameter range ~0.8–1.2 nm and individual length range ~100–1000 nm. Water was purified from a Millipore system with a resistivity of 18 MΩ cm. DNase, RNase, protease-free, filtered, and autoclaved DNA-grade water from Fisher Scientific (Pittsburgh, PA) were also used in different experiments.

The gold electrode consisted of float glass covered with a 50-Å-thick titanium layer, and then a 100-Å-thick layer of 99.9 % pure gold that was obtained from Evaporated Metal Films Corporation (Ithaca, NY). Hydrophilic, isopore polycarbonate filter membranes with 0.1-μm pore sizes were obtained from Millipore (Billerica, MA). The NAP5 column for DNA purification was received from GE Healthcare Bio-Sciences (Pittsburgh, PA) and was stored at 4 °C before use.

### Surface Functionalization

The cold-coated glass surfaces were cleaned before proceeding to surface modification. The gold surfaces were cut into their required sizes for different characterizations and were placed in piranha solution (3:1, *v*/*v* H_2_SO_4_:H_2_O_2_) (caution: piranha solution is a strong oxidizing agent and should be handled with extreme care) for at least 2 h at room temperature for cleaning, and were then rinsed thoroughly with deionized water. The gold surfaces were then dried under a stream of argon gas before being placed for 24 h in a 0.5 mM cysteamine and 0.5 mM 2-ME solution in absolute ethanol to form surfaces with amine functionality. After monolayer attachment, the surfaces were rinsed thoroughly with ethanol and dried under the argon stream ready for SWCNT attachment in the next step.

### SWCNT Attachment to Gold Surfaces

Initially, SWCNTs were shortened by a previously published procedure that also generated -COOH groups at the nanotube ends [20]. Briefly, SWCNTs were placed in a 3:1 (*v*/*v*) H_2_SO_4_:HNO_3_ solution and sonicated for 8 h in a Branson 2510 sonication water bath at 70 °C. Upon removal from the sonication bath, the acid solution of the SWCNTs was diluted with Millipore water to approximately 10 % of the original concentration. After allowing the SWCNTs to settle, the supernatant was drawn off, and the remaining SWCNT solution was filtered through a VCTP-type filter membrane (EMD Millipore) under suction while adding water to the filtering SWCNTs until the filtrate pH reached approximately 7. Several filter membranes with SWCNTs were placed in *N*,*N*-dimethylformamide (DMF) and sonicated for 15 min to remove all the SWCNTs from the membranes, generating a black solution. The filter membranes were then removed from the solution, and dicyclohexlycarbodiimide (DCC) was added to the DMF and SWCNT solution to achieve a final concentration of 0.5 mg DCC and 0.2 mg/mL of SWCNTs. The solution was then poured over the previously prepared gold surfaces with cysteamine and 2-ME monolayers and was finally left for 20–24 h in the solution. This step was necessary to attach the shortened SWCNTs through amide bond formation between the free -COOH groups present at their ends and the amine-terminated cysteamine monolayers [6]. The surfaces were then removed and placed in fresh DMF and sonicated for 1 min to remove any non-covalently bonded SWCNTs. Finally, the surfaces were rinsed thoroughly with Millipore water, dried under an argon stream, and used immediately for probe-DNA attachment.

### Probe-DNA Attachment to SWCNT Arrays

Gold surfaces with covalently attached SWCNTs were placed in a DMF solution of DCC with a concentration of 0.5 mg/mL for 2 h to prime the SWCNTs for amide bond formation before being rinsed with Millipore water, dried under an argon stream, and placed in a DNA attachment buffer solution of 10 μM probe-DNA [21]. The DNA attachment buffer contained a probe-DNA, 0.3 M NaCl, and 0.1 M NaHCO_3_ (pH 8.3) and was made with DNA-grade water. Surfaces were incubated in the DNA attachment buffer for 20–24 h at room temperature, then rinsed with DNA-grade water, dried under an argon stream, and either used immediately or stored at 4 °C for future use.

### Probe-DNA Attachment Directly to Gold Surfaces

The probe-DNA used in this case was connected to a small molecule through a disulfide bond that needed to cleave in order to generate a free thiol group at the 5′ end. This step was done by incubating the probe-DNA with 50 mM dl-dithiothreitol (DTT) solution for 4 h. Argon was bubbled through the DTT solution prior to the addition of the probe-DNA in order to remove oxygen from the solution to avoid aerial oxidation of DTT. To separate the small molecule from the probe-DNA, the solution was passed through a NAP5 column using the DNA attachment buffer. This was done by first allowing the temperature of the NAP5 column to reach room temperature. Subsequently, 2.5 mL of the DNA attachment buffer was allowed to enter the NAP5 gel bed completely, and this was carried out three times to equilibrate the column. Then, 90 μL of the probe-DNA in DTT solution was added, followed by 410 μL of the DNA attachment buffer, and was allowed to enter the gel bed completely. Finally, the probe-DNA was eluted into five separate aliquots of 200 μL each in microcentrifuge tubes. The probe-DNA was quantified using a NanoDrop2000 (Thermo Scientific, Wilmington, DE) ultraviolet–visible (UV–Vis) spectrophotometer by measuring absorption at a wavelength of 260 nm. For the probe attachment, clean gold surfaces were then incubated in 1 μM DNA solution made from the DNA attachment buffer for 18 h. Upon removal from the solution, the surfaces were rinsed with DNA-grade water, dried under an argon stream, and placed in a 1 mM 2-ME solution for 1 h. Finally, the gold electrodes were rinsed with DNA-grade water, dried under an argon stream, and either used immediately or stored at 4 °C for future use.

### Electrochemical Measurements

Impedance and CV measurements were conducted in a 3-mL maximum working volume, Teflon® electrochemical cell employing a standard three-electrode configuration controlled by a CHI660C electrochemical workstation (CH Instruments, Inc., Austin, TX). An aqueous Ag/AgCl, NaCl (3 M) was used as the reference electrode, a 99.99 % pure coiled platinum wire with a diameter of 0.25 mm served as the counter electrode, and the gold surfaces were used as the working electrodes. Impedance measurements were performed at the formal potential of the redox couple with an AC amplitude of 5 mV over a frequency range of 100 kHz to 0.1 Hz. The solution used for impedance measurements always contained equal concentrations of the oxidized and reduced forms of the redox couple: for example, 1 mM of both K_3_Fe(CN)_6_ and K_4_Fe(CN)_6_ in 1.0 M KCl. All solutions for electrochemical measurements were made with DNA-grade water. The formal redox potential [*E*_½_ = (*E*_p_^c^ + *E*_p_^a^)/2], where [Fe(CN)_6_]^3−^ = [Fe(CN)_6_]^4−^, was determined using cyclic voltammetry. For all hybridization assays, 300 nM concentrations of complementary or non-complementary DNA were used.

Impedance analysis was carried out using the commercially available Windows-based ZSimpWin®3.21 computer program, which determines the parameters of the assumed equivalent circuit by a nonlinear least-squares fit. All experiments were conducted at room temperature (25 ± 0.1 °C). A modified Randles [22] equivalent circuit (*vide infra*) used to fit the experimental impedance data comprised the solution resistance (*R*_s_), a constant phase element (*Q*_dl_) (used here as a generalized capacitance) in parallel with a resistance *R*_ct_ (the charge-transfer resistance), and a Warburg element (*W*, related to a diffusion-controlled process) in series. In admittance representation, a constant phase element is defined as *Y*(*ω*) = *Y*_*o*_ (*jω*)^*n*^, where *Y*_*o*_ and *n* are adjustable parameters (*n* = 0,1 corresponds to an ideal resistor and a capacitor, respectively), and *ω* is the angular frequency. The *R*_ct_ values can also be directly obtained from the diameter of the high-frequency “semicircle”.

### Characterization of VASWCNT Arrays

Atomic force microscopy (AFM) imaging was performed using a Veeco (now Bruker Instruments) (Santa Barbara, CA) multimode system, equipped with a Nanoscope IIIa controller, in tapping mode. The cantilevers were phosphorous-doped silicon and were specific for tapping mode imaging. The local root-mean-squared (rms) surface roughness was determined using height data from at least four representative 5 μm × 5 μm scan areas through the roughness analysis program included in the AFM analysis software. AFM imaging was performed on bare gold-coated glass surfaces as well as gold surfaces after covalent attachment of acid-shortened carbon nanotubes from solution.

## Results and Discussion

**Fig. 1:**
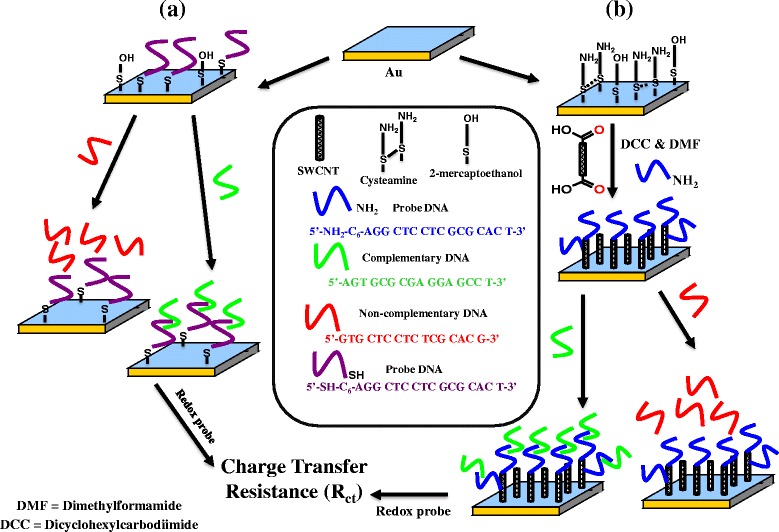
Schematic of DNA hybridization on gold and SWCNT-functionalized surfaces. **a** Surface attachment of probe-DNA with a thiol group at the 5′ end directly to the gold-coated glass surface and subsequent hybridization using cDNA and nCDNA. 2-ME is used as component in surface functionalization to prevent any possible nonspecific adsorption of probe-DNA. **b** Attachment of probe-DNA with an amine group at the 5′ end to the -COOH-functionalized VASWCNTs on the cysteamine/2-ME-modified gold surface and corresponding hybridization using cDNA and nCDNA

In the present study, two different modes of single-stranded probe-DNA attachment are followed; these are illustrated in Fig. 1 (a and b). In one case, probe-DNA attachment directly to the gold surfaces is achieved using a thiol-terminated probe-DNA using a strong gold–thiol interaction (Fig. 1a). In this case, the surfaces are further modified using 2-ME to prevent any possible nonspecific interactions during hybridization. In the other case, VASWCNTs on the amine-terminated gold surface are created using a self-assembled monolayer of aminothiol. A mixed monolayer of cysteamine and 2-ME (Fig. 1b) is used to prevent any nonspecific adsorption and horizontal alignment of shortened SWCNTs on the surface [23]. Sonication of SWCNTs in a 3:1 (*v*/*v*) solution of concentrated H_2_SO_4_ (98 %) and concentrated HNO_3_ (70 %) is carried out, resulting in short carbon nanotubes with carboxylic acid functionalities at the ends and at the defect sites of the walls [20]. These carboxylic acid functionalities on the SWCNTs are used to covalently link them to the amine-terminated gold surfaces in the presence of DCC as the coupling agent in DMF solution (Fig. 1b) [11].

The coupling of the single-stranded probe-DNA to VASWCNTS is achieved by coupling the amine-terminated probe-DNA in the presence of DCC in DMF solution with carboxylic acid-functionalized shortened SWCNTs, followed by hybridization of the immobilized probe to specific complementary DNA (cDNA) sequences in solution. In both cases, the subsequent detection of DNA hybridization relies on DNA duplex formation, with the immobilized single-stranded probe-DNA attached to the transducer surface, which converts the surface binding event to a useful electrical response. EIS is used to detect the changes in surface charge at the electrode–solution interface due to DNA hybridization. The measurements are based on the response of an electrochemical cell to a small-amplitude perturbing sinusoidal voltage signal. The resulting current response is used to calculate impedance from the ratio of the system voltage response to the current response. The complex impedance can be presented as the sum of the real (Z′) and imaginary (Z″) components that originate mainly from the resistance and capacitance of the cell, respectively. Since DNA is anionic, the formation of a DNA duplex on the electrode surface will generate an increasing negatively charged interface that will electrostatically repel an anionic redox probe such as [Fe(CN)_6_]^3−/4−^ in solution. The electrostatic repulsion of the redox probe from the electrode introduces a barrier for electron transfer, which is expected to show an increase in the interfacial electron transfer resistance, and will be reflected in the Z′ vs Z″ plot [24–26].

**Fig. 2:**
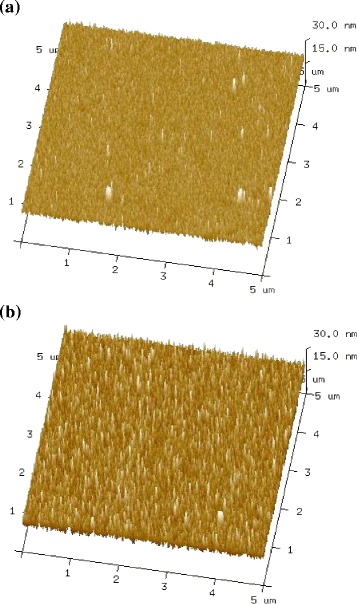
AFM images. Three-dimensional tapping mode 5 μm × 5 μm height image of **a** the bare gold-coated glass surface and **b** VASWCNT arrays generated on a mixed monolayer of cysteamine/2-ME on the gold surface after 24 h incubation. *Z*-scale is the same for **a** and **b**

AFM is used to characterize the nanotube arrays to ensure that the SWCNTs are bound in a perpendicular orientation rather than lying flat on the surface. Compared with the AFM image of a bare gold surface in Fig. 2a, the surface after SWCNT attachment (Fig. 2b) appears to be much rougher. Bundles of densely packed, needle-like patterns of standing SWCNTs are observed with a maximum length of ~30 nm after 24 h of coupling on the cysteamine/2-ME mixed monolayer surface. It is to be noted that the horizontal length of acid-shortened SWCNTs after 8 h of sonication measured by AFM imaging provides a bimodal distribution with peaks at ~50–60 nm and ~120–130 nm. Comparing height of the nanotubes attached to the surface, it is evident that only relatively short nanotubes can be immobilized on the gold surface through condensation reaction [6]. A gradual increase in the surface coverage of SWCNTs has also been observed (data not shown) with increased coupling time [11]. This geometry of the surface attachment can be explained by taking into account the high concentration of the carboxylic acid groups generated at the ends of the shortened nanotubes, which are capable of forming a number of covalent bonds with the amino groups on the gold surfaces and of forming strong hydrophobic interactions between adjacent SWCNTs. Further evidence of the surface attachment of SWCNTs comes from measuring the rms surface roughness values of specific surfaces from their corresponding AFM height images. Specifically, analysis of a number of 5 μm × 5 μm AFM images shows that the surface roughness increases from 1.17 ± 0.02 nm for a bare gold surface to 2.07 ± 0.03 nm after SWCNT attachment. A similar order of magnitude increase in the surface roughness has also been observed in a previous study [4] involving generation of VASWCNTs, confirming the alignment of SWCNTs perpendicular to the surface. It should be noted that there exists a tip convolution problem in AFM and that it is known that the height measured using AFM is always lower than the length of the free carbon nanotubes adsorbed on the surface [23].

**Fig. 3:**
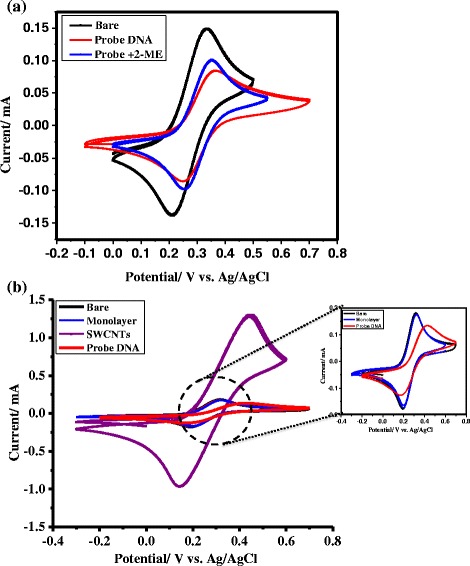
Cyclic voltammetric responses in different steps of surface functionalization. Cyclic voltammetric responses representing different steps in attaching **a** probe-DNA directly to the gold surface and **b** probe-DNA to VASWCNTs on the gold surface in 1 mM [Fe(CN)_6_]^3−/4−^ + 0.1 M aqueous KCl solution. The *inset* shows a closer view of the cyclic voltammograms in different steps except with VASWCNTs. Scan rate = 50 mV/s and electrode area = 2.67 cm^2^. In all measurements, aqueous Ag/AgCl is used as reference electrode

**Fig. 4:**
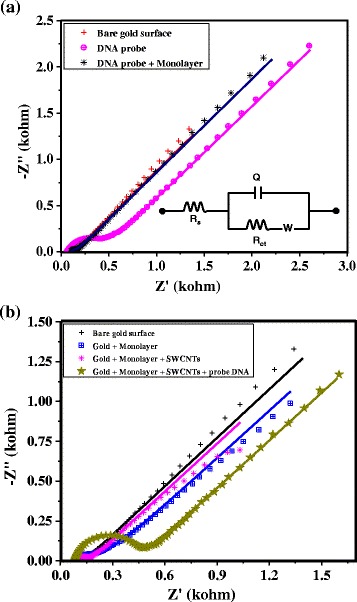
Impedance responses of different steps of probe-DNA attachment. Complex impedance plot at a DC bias of +0.25 V vs Ag/AgCl in 1 mM [Fe(CN)_6_]^3−/4−^ + 0.1 M aqueous KCl solution for **a** different steps of probe-DNA attachment directly to the gold surface and **b** different steps of probe-DNA attachment to VASWCNTs on the gold surface. The frequency range is 100 kHz to 0.1 Hz with 5 mV rms signal. *Solid lines* are the fit to the experimental response using an appropriate equivalent circuit, which is shown as an *inset* of **a**. See the “Methods”/“Materials” section of the text for a more detailed discussion about the specific components of the equivalent circuit used here

In the present work, we use CV and impedance measurements to detect changes at the electrode–solution interface during different steps of surface preparation in two different modes of probe attachment, as well as the subsequent detection of hybridization. Electrochemical measurements show appreciable changes in CV (Fig. 3) and impedance (Fig. 4) responses during different stages of surface modification for both modes of probe attachment, measured in the presence of the Fe(CN)_6_^3−/4−^ redox probe in 0.1 M KCl solution. Specifically, Fig. 3a illustrates the superimposed CV responses of bare gold surfaces after mixed monolayer formation and probe-DNA attachment directly to the gold surfaces. It is evident that the peak current associated with the Fe(CN)_6_^3−/4−^ redox response decreases after monolayer formation due to partial blocking of electron transfer at the electrode surface. It is apparent that the mixed monolayer used in this case contains a number of defect sites (pinholes) through which the probe molecules can permeate completely or partially. Upon probe-DNA attachment, the peak current due to the Fe(CN)_6_^3−/4−^ redox probe becomes further suppressed due to repulsion of the negatively charged redox probe by the anionic probe-DNA attached to the surface. Figure 3b shows the CV response in different steps of the surface functionalization when the probe-DNA is attached to SWCNTs. Similar to the previous case, a mixed monolayer of cysteamine/2-ME provides essentially no barrier to electron transfer of the probe molecule compared to a bare gold surface. Interestingly, a significant increase in the peak current is observed for the electrode with SWCNTs compared to the monolayer-coated response. This behavior suggests an electron transfer mediator role of aligned SWCNTs between the gold electrode and the redox probe in solution. It is interesting to note that although peak current has increased upon SWCNT attachment to the gold surface compared to the mixed monolayer step, the redox peak separation has also increased for the SWCNT-modified electrode (Fig. 3b). As we have used cysteamine as the underlying organic monolayer, it provides a monolayer with lower packing density compared to a monolayer of long-chain amine used in a previous study [13]. In effect, surface density of tethered SWCNTs will also be less in our case compared to a monolayer with long alkyl chain. It is also known that redox peak separation decreases with increase in surface density of SWCNTs which in turn is responsible for decrease in apparent tunneling resistance. So, the observed increase in peak separation after SWCNT attachment in our case is presumably due to lack of high surface coverage of surface-bound SWCNTs which can possibly be achieved using long-chain amino thiols. This scenario may create an ohmic drop leading to higher peak potential separation which can be compensated with higher surface density SWCNTs. Eventually, the peak current drastically decreases once the probe-DNA is attached to the SWCNTs due to the repulsive interaction of the surface-bound anionic probe-DNA with the negatively charged redox probe in solution (see inset of Fig. 3b for a closer view).

Impedance responses in the form of Nyquist plots for various stages of surface functionalization depicted in Fig. 1 are shown in Fig. 4a, b for both modes of probe attachment. These impedance responses are fitted with the equivalent circuit shown in the inset of Fig. 4a to extract the appropriate circuit parameter to assess the surface binding events. For probe-DNA attachment directly to the gold surface, Fig. 4a shows superimposed impedance responses at the different stages of surface modification. We focus our attention on the more interesting part of the spectrum at the higher frequencies where the electrode reaction is purely kinetically controlled and the heterogeneous charge-transfer resistance (*R*_ct_) is expected to increase due to possible inhibition of electron transfer at the electrode–solution interface. It is known that at higher frequencies, the diameter of the semicircle corresponds to the *R*_ct_ of the monolayer-coated electrode. Comparison of the complex impedance plots in Fig. 4a shows that the semicircle measured at the bare electrode is poorly defined and that no significant change in *R*_ct_ values is observed due to mixed monolayer formation. These results indicate that the electron transfer is fast and the monolayer-coated electrode fails to block the electron transfer of the redox probe in solution. However, significant changes in the *R*_ct_ values are observed between the monolayer-coated electrode (~61.33 Ω) and the electrode with the attached probe-DNA (~422.1 Ω), which can be attributed to the repulsion of the negatively charged redox probe by the anionic probe-DNA attached to the VASWCNTs. It should be noted that these results follow a similar trend to that observed for the CV data in Fig. 3a. Impedance results obtained from various steps of surface functionalization with the probe-DNA attached to the VASWCNT electrodes are shown in Fig. 4b. Similar to the results obtained in the case of probe-DNA attachment directly to the gold surfaces, no significant changes in *R*_ct_ values are observed between the bare gold surfaces and the monolayer-coated gold surfaces. The *R*_ct_ value has further decreases at the SWCNT-modified electrode, suggesting faster electrode kinetics of the redox probe after SWCNT attachment. These results corroborate the results shown in Fig. 3b with significant increases in redox current at the SWCNT-modified electrode, suggesting that the SWCNTs play the role of mediator of electron transfer at the electrode–solution interface. Further modification of SWCNTs with the probe-DNA shows a significant increase in *R*_ct_ values (~365.3 Ω), as is evident from the high-frequency semicircle conforming to the successful probe-DNA attachment. A significant decrease in redox current is also observed in the corresponding previous CV response in Fig. 3b at the same stage of surface modification.

**Fig. 5:**
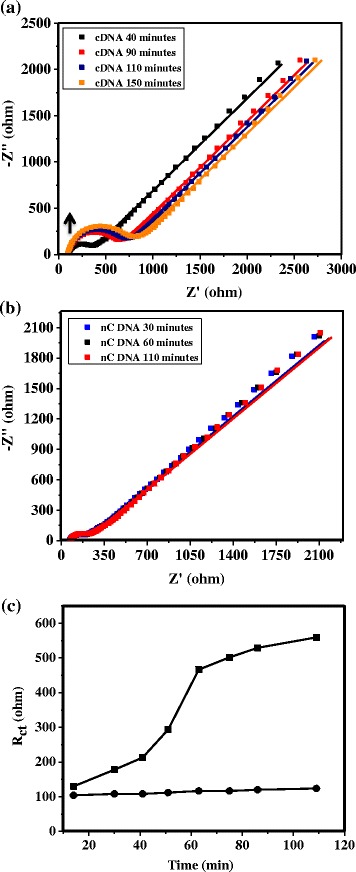
Impedance responses during hybridization of probe-DNA attached directly to the gold surface. Time-dependent impedance response of probe-DNA attached to the gold surface in 1 mM [Fe(CN)_6_]^3−/4−^ + 0.1 M aqueous KCl with **a** 300 nM cDNA and **b** 300 nM nCDNA, and **c** variation of charge-transfer resistance (*R*_ct_) against time for cDNA (*black squares*) and nCDNA (*black circles*). An *upward arrow* in **a** indicates increase of *R*_ct_ (diameter of semicircles) with time. *Solid lines* are the fit to the experimental response using an appropriate equivalent circuit, which is shown as an *inset* of Fig. 4 **a**

**Fig. 6:**
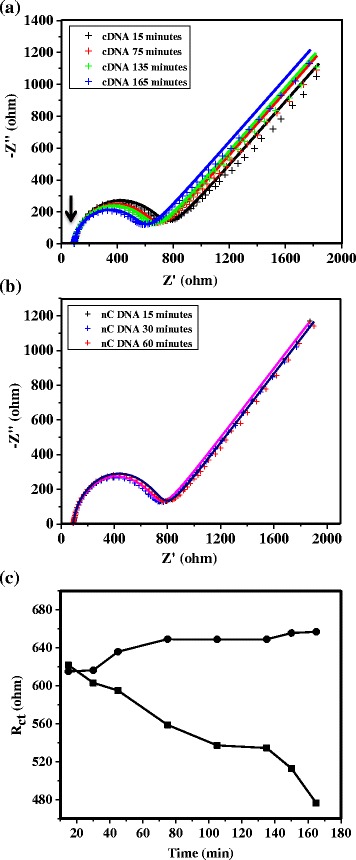
Impedance responses during hybridization of probe-DNA attached to VASWCNTs on the gold surface. Time-dependent impedance response of probe-DNA attached to SWCNTs on the gold surface in 1 mM [Fe(CN)_6_]^3−/4−^ + 0.1 M aqueous KCl with **a** 300 nM cDNA and **b** 300 nM nCDNA, and **c** variation of charge-transfer resistance (*R*_ct_) against time for cDNA (*black squares*) and nCDNA (*black circles*). A *downward arrow* in **a** indicated decrease of *R*_ct_ (diameter of semicircles) with time. *Solid lines* are the fit to the experimental response using an appropriate equivalent circuit, which is shown as an *inset* of Fig. 4 **a**

**Fig. 7:**
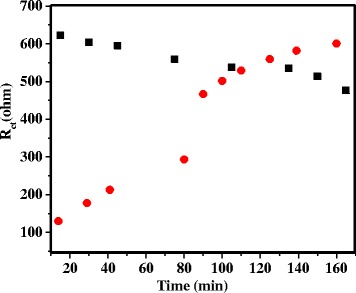
Variation of charge-transfer resistance with time. Comparative change in charge-transfer resistance with time measured from impedance response during hybridization of cDNA to probe-DNA attached directly to the gold surface (*red circle*) and probe-DNA attached to VASWCNTs on the gold surface (*black square*). Impedance measurements are done in the presence of 1 mM [Fe(CN)_6_]^3−/4−^ and 0.1 M aqueous KCl solution

We have further performed DNA hybridization using Fe(CN)_6_^3−/4−^ as the redox probe in the presence of 300 nM cDNA or non-complementary DNA (nCDNA) in 0.1 M KCl solution, and results of the time-dependent impedance responses of DNA hybridization are shown in Fig. 5, with the probe-DNA attached directly to the gold surfaces. It is evident that *R*_ct_ in the form of the high-frequency semicircle has increased with time during DNA hybridization (Fig. 5a), following a similar trend reported in the literature [24, 26], with no such changes observed for nCDNA with time (Fig. 5b). A combined plot of changes in *R*_ct_ with time for both cases is shown in Fig. 5c. The increase in *R*_ct_ with time in this case is due to the increased repulsion of the negatively charged redox probe by an increased number of anionic DNA on the surface with time due to hybridization. In contrast, a decrease in *R*_ct_ is observed for cDNA (Fig. 6a) with time, while no such changes are observed for nCDNA (Fig. 6b) in solution during hybridization when the probe-DNA is attached to VASWCNTs. The changes in *R*_ct_ with time for cDNA and nCDNA are plotted together in Fig. 6c. A relatively rapid increase in *R*_ct_ is observed for DNA hybridization with the probe-DNA attached directly to the gold surfaces compared to a slow decrease of *R*_ct_ with time when the probe-DNA is attached to VASWCNTs, which is evident from the combined plot of *R*_ct_ against time in Fig. 7.

Considering the electrochemical responses of both the interfaces with the probe-DNA attached to VASWCNTs and directly to the gold surfaces, it is obvious that these systems do not form a passivating interface, as significant permeation of the redox probe is observed, which is evident from the prominent Warburg part in the impedance response at the low frequencies in Fig. 4. Such ion permeation at the sensing interface can be advantageous for recognition of DNA hybridization through modulation of the charge-transfer resistance due to DNA binding events. Increases in charge-transfer resistance during hybridization of the probe-DNA attached directly to gold follow the expected trend reported in the literature considering the repulsion of the negatively charged Fe(CN)_6_^3−/4−^ probe by the increased concentration of anionic DNA with time due to hybridization. However, the decrease in charge-transfer resistance during hybridization when the probe-DNA is attached to VASWCNTs requires further discussion. There are a number of earlier reports [17, 27] where a decrease in charge-transfer resistance is attributed to the possible morphological changes going from single-stranded to double-stranded DNA during hybridization. It is presumed that double-stranded DNA, produced during hybridization, is stiffer and also more hydrophilic than single-stranded DNA. It is also more orthogonal and is further away from the surface to facilitate access of the redox probe to the electrode where charge transfer is efficient. This anomalous decrease in charge-transfer resistance can also be rationalized by the “ion gating” effect when considering a single-stranded DNA as a flexible molecule as opposed to a “rigid rod”-like structure for the double-stranded DNA. This process can open up the interface for the redox probe to access the electrode at the defect sites (pinholes) in the mixed monolayer. It is indeed intriguing that the presence of probe-DNA at the end of aligned SWCNTs provides a barrier of electron transfer to the redox probe in solution (Fig. 4b) but starts facilitating electron transfer during the course of hybridization (Fig. 6a). It is plausible that the single-stranded probe-DNA attached at the end of SWCNTs is flexible and lies across the interface providing effective electrostatic repulsion to the negatively charged redox probe resulting in increased charge transfer resistance (suppressed electron transfer). On the contrary, initiation of hybridization process generates a more rigid double-stranded structure with increased hydrophilic character. This situation provides comparatively more access to the redox probe through the interface leading to its close approach to SWCNTs causing an enhanced electron transfer [17, 27]. If the morphological changes in the DNA structure or the ion gating effect are indeed the possible reasons for the decrease in charge-transfer resistance, we would expect to observe a similar trend for the change in charge-transfer resistance during hybridization when the probe-DNA with the same base sequence is attached directly to the gold surfaces. Considering the results presented here and also those reported in the literature, it can be concluded that the decrease in charge-transfer resistance is due to VASWCNTs acting as a mediator of electron transfer between the redox probe in solution and the underlying electrode surface.

## Conclusions

In summary, VASWCNT arrays have been generated on gold surfaces and have shown significant changes in surface roughness compared to bare gold surfaces. Subsequently, these nanotube arrays have been functionalized with a single-stranded probe-DNA to follow the hybridization process by time-dependent impedance measurements in the presence of Fe(CN)_6_^3−/4−^ as the redox probe in solution. In addition, hybridization has also been followed with the probe-DNA attached directly to the bare gold surfaces. It has been found that the charge-transfer resistance decreases with time when the probe-DNA is attached to VASWCNTs but increases when the probe-DNA is attached directly to the gold surfaces. Impedance and CV measurements have been used to follow the changes in interfacial properties in the presence of Fe(CN)_6_^3−/4−^ during different steps of surface functionalization. The unexpected decrease in charge-transfer resistance during the progress of hybridization indicates mediated electron transfer by the VASWCNTs between the electrode and the redox probe in solution. The current methodology of DNA detection can further be extended to detect other biomolecules.

## Abbreviations

2-ME: 2-mercaptoethanol
cDNA: complementary DNA
DCC: dicyclohexlycarbodiimide
DMF: *N*,*N*-dimethylformamide
DTT: dl-dithiothreitol
EIS: electrochemical impedance spectroscopy
nCDNA: non-complementary DNA
SMA: self-assembled monolayer
SWCNTs: single-walled carbon nanotubes
VASWCNT: vertically aligned single-walled carbon nanotube
